# Supplementary material for: Leptospira interrogans Serovar Icterohaemorrhagiae Failed to Establish Distinct Infection in Naïve Gilts: Lessons Learned from a Preliminary Experimental Challenge
Source: Pathogens. 2023 Jan 13;12(1):135. doi: 10.3390/pathogens12010135 (PMC9867409; doi:10.3390/pathogens12010135)
Supplement: Supplementary file 1 [file pathogens-12-00135-s001.zip › pathogens-2148866-supplementary.pdf]

Table S1: Microscopic agglutination test (MAT) titers of infected gilts (no 6, 8 and 9) against *Leptospira* serovar Icterohaemorrhagiae (inoculated serovar) on ten study days.

**Study days**	**gilt no 6**	**gilt no 8**	**gilt no 9**
D0	0	0	0
D2	0	0	0
D4	100	50	0
D7	1600	800	100
D10	800	800	100
D14	800	1600	100
D17	800	800	100
D21	800	800	0
D24	800	800	0
D28	200	400	0
